# So you married a doctor? Preparing trainees for medical marriages

**Published:** 2013-03-31

**Authors:** Monica Lypson, Rachel Perlman, Paula Ross

**Affiliations:** 1 Office of Graduate Medical Education, University of Michigan Medical School, Ann Arbor, MI; 2 Department of Internal Medicine, University of Michigan Medical School, Ann Arbor, MI; 3 Office of Medical Student Education, University of Michigan Medical School, Ann Arbor, MI

Most medical marriages occur before graduation from medical school; however, many medical students are unaware of the unique challenges associated with careers in medicine (e.g., financial insecurity, fatigue, stress, academic trouble, etc.) as they prepare to merge their careers with marriage or other long term relationships.^1^ Physician relationships in both the United States and Canada typically mirror national relationship trends in the larger population with a few exceptions.^2^–^4^ For example, physicians entering marriage tend to be older and divorce rates may be gender specific; the divorce rate for female physicians is higher than male physicians, the marriage rate is higher for female physicians than non-physician females, and approximately half of female physicians marry other physicians.^5^

Recently, both the Liaison Committee on Medical School and the Royal College of Physicians and Surgeons of Canada included requirements toward helping trainees better manage their health and wellness. In the United States the focus is on medical student wellness and programs to promote the well-being of medical students and facilitate their adjustment to the physical and emotional demands of medical education.^6^ The CanMEDS 2005 Framework, under the competency of “professional”, requires a commitment to physician health.^7^

Trainees remain curious about how medical marriages function in real life, including the challenges of balancing the demands of work, personal relationships and children; however they are typically provided with little insight to help guide them. Given that medical school is the time when many students are beginning marriages or other long term partnerships, we developed a workshop for medical students to explore these issues using an encouraging approach to highlight the positive aspects of relationships to promote trainee wellness through a focus on work-family balance in physician domestic partnerships.

The workshop explored existing literature in this area along with first-hand accounts from physician couples. We targeted topics such as managing the “Super Couple Syndrome”, the “Good Enough Mother” and identifying potential relationship threats. We also addressed protective actions such as managing stress, remaining engaged in the relationship, paying attention to parenting, and implementing communication methods that enhance friendship and intimacy. 8

Individuals from single- and dual-physician marriages as well as same-sex partnerships were invited to discuss their relationship experiences and the implications of medical marriages. They also discussed potential barriers and challenges to managing their own personal and professional lives. Invitees also answered questions addressing medical students’ concerns about the challenges and struggles that come from maintaining a relationship when starting a career in medicine, such as career decisions and the role of physicians in intimate relationships.

Our evaluations revealed that the trainees found the workshop useful in getting candid advice from real people who have experienced dual career domestic partnerships and also helped them recognize the importance of giving considerations to future work-family balance and potentially lifestyles that may be unique to certain specialties. Both faculty and trainees affirmed that it was critical to provide doctors-in-training with exposure to issues of work-family balance as they prepare for their future careers in medicine. Faculty participants also stated the workshop gave them the opportunity to reflect on their career path and appropriate work-life balance, and provided the opportunity to listen to their spouses’ perceptions and attitudes about their career and relationship.

Ultimately, the exposure to evidence-based information on work-life balance and the candid advice from real people who have “gone the course” helped trainees better recognize this area of physician wellness.
